# Performance of Elephant Herding Optimization and Tree Growth Algorithm Adapted for Node Localization in Wireless Sensor Networks

**DOI:** 10.3390/s19112515

**Published:** 2019-06-01

**Authors:** Ivana Strumberger, Miroslav Minovic, Milan Tuba, Nebojsa Bacanin

**Affiliations:** 1 Faculty of Informatics and Computing, Singidunum University, Danijelova 32, 11010 Belgrade, Serbia; istrumberger@singidunum.ac.rs (I.S.); tuba@ieee.org (M.T.); 2 Faculty of Organizational Sciences, University of Belgrade, Jove Ilica 154, 11010 Belgrade, Serbia; miroslav.minovic@fon.bg.ac.rs

**Keywords:** node localization, wireless sensor networks, swarm intelligence, elephant herding optimization, tree growth algorithm, NP hardness

## Abstract

Wireless sensor networks, as an emerging paradigm of networking and computing, have applications in diverse fields such as medicine, military, environmental control, climate forecasting, surveillance, etc. For successfully tackling the node localization problem, as one of the most significant challenges in this domain, many algorithms and metaheuristics have been proposed. By analyzing available modern literature sources, it can be seen that the swarm intelligence metaheuristics have obtained significant results in this domain. Research that is presented in this paper is aimed towards achieving further improvements in solving the wireless sensor networks localization problem by employing swarm intelligence. To accomplish this goal, we have improved basic versions of the tree growth algorithm and the elephant herding optimization swarm intelligence metaheuristics and applied them to solve the wireless sensor networks localization problem. In order to determine whether the improvements are accomplished, we have conducted empirical experiments on different sizes of sensor networks ranging from 25 to 150 target nodes, for which distance measurements are corrupted by Gaussian noise. Comparative analysis with other state-of-the-art swarm intelligence algorithms that have been already tested on the same problem instance, the butterfly optimization algorithm, the particle swarm optimization algorithm, and the firefly algorithm, is conducted. Simulation results indicate that our proposed algorithms can obtain more consistent and accurate locations of the unknown target nodes in wireless sensor networks topology than other approaches that have been proposed in the literature.

## 1. Introduction

As an emerging paradigm of networking and computing, wireless sensor networks (WSNs) have been relevant and applicable in diverse fields such as medicine, military, environmental control, climate forecasting, surveillance, etc. Consistent development and advances in networks have significantly extended and enabled broad application of WSNs. Recently, WSNs have been integrated with other concepts, like with the concept of the internet of things (IoT) [1].

A WSN is a network infrastructure that consists out of vast number of minuscule, diminutive, inexpensive autonomous devices denoted as sensor nodes, which monitor and detect the environment in order to compile data [2]. The data which is collected from the environment is afterwards sent to the sink node, a destination where data can be processed locally or redirected to other networks for different uses [3]. Due to their accessible deployment, node communication, data transfer, and self-organization, WSNs have many advances and usage, however, they also experience some challenges.

There are numerous challenges in WSNs’ implementation process, such as node localization, coverage, energy consumption of sensor nodes, data routing issues, etc. Despite all of these challenges and issues, the most significant one is determining the location of sensor nodes. In order for data to be effectively collected from the environment, the locations of all sensor nodes ought to be known and valid. If altered, the data cannot be relevant for other utilization and processes. Because of the terrain deployment and different outdoor climates and indoor environments, some sensor nodes cannot be reached, and for that problem there has to be a communication and connection structure where at each moment all sensor nodes are visible and obtained for collecting and transferring data for future use. There are some approaches that consider using global positioning system (GPS) for each sensor node that is deployed, but it can result in malfunction of the nodes, energy consumption for data transfer and node usage, as well as cost and size.

Considering these challenges and deployment structure in environment that is monitored, the preferred alternative is to localize deployed nodes. The WSNs localization problem can be defined as the process of finding exact locations of the unknown target sensor nodes that are randomly deployed in the monitoring environment by using the existing precise locations of sensor nodes, denoted as anchor nodes, which are utilized to obtain the distance and positions of target nodes with methods such are triangulation [4], time of arrival (ToA) [5], radio signal strength (RSS) [6,7,8], angle of arrival (AoA) [9], etc. In most cases, anchor nodes are equipped with GPS system and their location can be precisely determined. For this type of GPS and non-GPS sensor nodes, many localization algorithms and methods exist, and they can be classified as range-based and range-free approaches [10]. As its name implies, range-based localization algorithms utilize distance assessment and/or angle-based techniques between unknown target nodes and anchor nodes, whose positions are known. When the distance between unknown target nodes and anchor nodes is assessed, the triangulation (or trilateration) techniques can be applied to find coordinates of unknown target nodes. Range-free localization algorithms are based on topological data and could provide more precision in finding the positions of the target nodes. However, previously conducted research showed that these algorithms are not affordable and economic in practical implementations [11].

Since the WSNs are often deployed in indoor environments that contain walls, interference, multi-path effect, humidity and temperature variations, it is important to emphasize significance of node localization in such surroundings. In such scenarios, sensors could find their positions inside a floor of a building using wireless local area network (WLAN) technology [6]. In this area, an extensive research has been performed by the prominent authors, who have developed state-of-the-art approaches for self-location of wireless sensors in indoor environments [6,12,13].

The goal of node localization is to obtain geographical coordinates for all distributed sensor nodes which locations are unknown in the monitoring environment. Following [14,15], the problem which is considered in these papers consists of WSN with sensor nodes randomly deployed in two-dimensional (2*D*) monitoring environment. Each distributed sensor node has a transmission range *r*. The WSN can be defined as an Euclidean graph G=(V,E), where *V* represents the group of sensor nodes (i,j)∈E, while the distance between the *i*-th and the *j*-th sensor node is *r*. Unknown target nodes are denoted as the set of *M*, and localized nodes, whose positions are acquired with localization technique, are represented as the set of *L*. Anchor nodes are defined as set *N*, with positions ($x_{n},y_{n}$), for all n∈N. The goal of the localization problem is to find the positions ($x_{m},y_{m}$) of as many as possible target nodes m∈M, where unknown target nodes are resolved into a localized *L* set of nodes.

The node localization problem belongs to the group of NP-hard optimization, due to its complexity and many instances [3,16]. Since deterministic algorithms could not solve NP hard optimization problems within reasonable computational time, non-deterministic (stochastic), metaheuristics methods can be employed. Despite of the fact that the optimal solution could not be assured, when implemented, metaheuristics methods can achieve a suboptimal acceptable solution within a suitable time-frame.

As a subgroup of metaheuristics algorithms, swarm intelligence approaches simulate groups of wide variety of organisms from the nature, such are flock of birds, hives of bees, colonies of ants, groups of bats, herds of elephants, etc., by following natural principles. These natural principles are based on group intellectual communication not on individual, because the measure of the intellectual resourcefulness is seen through group unification, not as an individual entity. Swarm intelligence metaheuristic algorithms are population-based and iterative search methods that utilize positive feedback, negative feedback, multiple interactions, and fluctuation among individuals in the search process [17].

By analyzing available sources from the literature, it can be seen that the swarm algorithms are able to obtain promising results in the domain of WSNs node localization [15,16,18]. The research that is presented in this paper is aimed towards achieving further improvements in solving the WSNs localization problem by applying swarm intelligence algorithms. This also represents the basic focus of this paper.

To achieve further improvements in unknown sensor nodes localization in WSNs topology, in terms of localization accuracy and precision, we have improved existing versions (implementations) of the tree growth algorithm (TGA) and the elephant herding optimization (EHO) swarm intelligence metaheuristics (EHO and TGA metaheuristics were devised by Wang and Cheraghalipour, respectively, and as such they existed prior to our version of these approaches). We have applied proposed improved swarm algorithms in solving the node localization problem in WSNs and enhanced results that have been previously obtained by other swarm intelligence approaches that have been tested on the same localization problem instance. When tackling node localization, we have included a multi-stage localization approach, which indicates that all unknown sensor devices are localized in multiple stages of the algorithm’s execution.

According to the literature survey, there are no previous implementations of any TGA based algorithm for the node localization problem in WSNs, while the original EHO has been previously adapted for tackling similar (not the same) problem instances [4,19].

The scientific contribution of this paper is twofold: improvements in solving the WSNs localization problem are established and the original versions of the TGA and EHO swarm intelligence metaheuristics are improved.

According to the above statements, the basic research question that is addressed in this paper can be formulated as follows: “Is it possible to achieve further improvements in solving the WSNs localization problem by applying swarm intelligence metaheuristics?”.

The details of all algorithm’s implementations, as well as the details of the control parameters’ adjustments and experimental conditions, are fully provided in this paper, so the researcher who wants to implement proposed approaches and to run simulations has more than enough information to do this on her/his own.

The rest of the paper is organized as follows. In Section 2 we have secluded a review of swarm intelligence metaheuristics and its applications in the domain of WSNs. Section 3 presents mathematical formulation of the node localization problem in WSNs that was utilized in the research conducted for the purpose of this paper. Section 4 presents the original TGA version, and our improved version adjusted for the node localization problem. In Section 5, we have presented details of the original and hybridized EHO algorithms for node localization. Conducted empirical simulations and obtained results, along with full details of the experimental setup, are shown and depicted in Section 6, which is followed by the final Section 7, which concludes this paper, and provides remarks and references for future research and work.

## 2. Review of Swarm Intelligence Metaheuristics for Wireless Sensor Networks

In this section we present literature review of swarm intelligence approaches for node localization in WSNs. Due to its importance on solving NP hard problems, at the beginning of this section we also show brief review of genetic algorithms application for this problem.

Real life problems such as WSNs node localization, transportation problems, portfolio optimization, can be modeled as optimization tasks. Optimization presents one of the most researched domains. Metaheuristic algorithms are widely implemented for practical NP hard problems, as can be seen in the literature.

Metaheuristics inspired by the nature can be in general divided into two groups: evolutionary algorithms (EA) and swarm intelligence. One of the most well-known representatives of EA is genetic algorithm (GA). GA proved to be capable of solving large number of NP hard problems, including problems from the domain of WSNs.

In Ref. [20], the authors proposed a distributed range-free node localization algorithm for three- dimensional WSNs based on the GA. Similarly, in Ref. [21], by applying the localization algorithm that employs GA, the localization accuracy of unknown nodes in WSNs was improved. Also, recently, a novel range free localization algorithm based on GA and connectivity was proposed [22].

One of the first swarm intelligence approaches that has been presented in the literature was particle swarm optimization (PSO) [23]. PSO metaheuristics conducts the search process by simulating flocks of birds and fish. This metheuristics has been widely applied to the domain of node localization in WSNs. In Ref. [24] the authors have proposed optimal parameter selection for nine PSO variants and six types of swarm topologies for tackling the WSNs localization problem. Besides those mentioned, there are also many other PSO implementations for node localization problems, for example, in Ref. [25] velocity adaptation based PSO for this problem was proposed, while in Ref. [26] localization accuracy was significantly improved by using a localization algorithm based on the PSO metaheuristics. Besides those mentioned, there are also hybridized PSO implementations for this problem [27]. In the literature survey, there are also variants of the PSO metaheuristics adapted for sink node localization. For example, in Ref. [28], the authors presented adaptive PSO (APSO) approach for determining the best location for a sink node in WSN topology.

One of the swarm intelligence metaheuristics with most implementations for various NP-hard optimization tasks is artificial bee colony (ABC), which has many applications for both, benchmark [29], and practical tasks [30,31]. According to the literature survey, many implementations of the ABC metaheuristics in the domain of WSNs localization can be found [32,33]. Firefly algorithm (FA) is another well-known swarm algorithms representative, which mimics the lighting properties of fireflies. According to the results presented in the literature, FA has robust metaheuristics for tackling various kinds of NP hard optimization problems in modified [34,35] and hybridized versions [36]. The FA approach has many applications to node localization of WSNs [15,37]. For example, in Ref. [38], the authors proposed implementation of the FA for a concept of projecting virtual anchor nodes for moving target node localization. Obtained results of the FA metaheuristics were compared with the results achieved by biogeography based optimization (BBO), PSO, and h-best PSO.

Monarch butterfly optmization (MBO) is a relatively novel swarm algorithms approach that was proposed in 2015 by Wang and Deb [39]. In this very first paper, the MBO’s creators proved that this metaheuristic has huge potential in dealing with NP-hard tasks. The MBO metaheuristics also has its implementations for the localization problem in WSNs, where it obtained promising results. For example, in Ref. [40], MBO was employed in multi-stage localization approach, by localizing all nodes in multiple stages of algorithm’s execution. A similar approach, named butterfly optimization algorithm (BOA), was also adapted for node localization [15]. Moth search (MS) algorithm is another relatively new swarm intelligence metaheuristics that was inspired by the phototaxis and Lévy flights of the moths. This metaheuristics was proposed in 2016 by Wang [41]. MS proved to be state-of-the-art method for tackling global optimization benchmark problems [41], as well as real world problems like drone placement problem [42] and WSN node localization [43].

Cuckoo search (CS) algorithm, which was inspired by the obligate brood parasitism of some cuckoo species, was developed in 2009 by Yang and Deb [44]. This swarm intelligence approach proved to be capable of solving variety of NP hard optimization problems [45]. Adaptations of CS for node localization can also be found in the literature survey [16]. In Ref. [18], an effective CS algorithm for node localization was proposed and an extensive experimental study, which measures the effects of parameters such as node and anchor density and communication range with respect to average localization error and success ratio, is shown. Another prominent representative of swarm intelligence is bat algorithm (BA), which has many successful applications to benchmark [46] and real world problems [47]. The BA approach was also applied to node localization [48].

In the review section of this paper it is also worth mentioning that the swarm algorithms were also applied to other problems from the WSNs domain. For example, in Ref. [49], a pigeon-based self-deployment algorithm (PSA) was proposed for underwater WSN with the aim of overcoming limitations of the existing algorithms. In Ref. [50], a low energy PSO approach was utilized for node location in optical WSN. The authors considered the impact of node position on energy consumption during the communication process over the network. PSO metaheuristics was also used for maximization of a WSN network lifetime [51]. To address routing problems in heterogeneous WSNs environments, a novel PSO based approach was presented in Ref. [52]. Also, in the domain of clustering WSN, many swarm intelligence algorithms were applied. Comprehensive literature review of optimization clustering algorithms for large WSNs can be found in Ref. [53].

## 3. Problem Statement

In this section of the paper we give mathematical formulation of the node localization problem instance that we utilized in the conducted research.

The objective of node localization in WSNs is the assessment of coordinates of the unknown target sensor nodes randomly distributed in the monitoring environment, with the goal to minimize the objective function. The evaluation of target node position is concluded with the range-based distributed localization technique, which is performed in two phases: the ranging phase and the position estimation phase.

For assessing the distance between target nodes and anchor nodes in the first phase, the intensity of the received signal was considered. Due to the fact that the signal is corrupted by Gaussian noise, the precise measurements could not be obtained. In the second phase, positions of target nodes are estimated by using geometric approach, trilateration method. The position estimation phase uses the previous obtained information from the ranging phase. Many different techniques for calculating the positions (coordinates) of target nodes exist, however in this particular problem statement, the trilateration method was utilized. Due to the measurement imprecision in these two phases, swarm intelligence algorithms can be implemented to minimize the localization error.

In a two-dimensional (2*D*) WSN monitoring environment, *M* target sensor nodes and *N* anchor nodes are randomly deployed, with the transmission range *R*. Measured distance between each target and anchor node are revised with the Gaussian noise variable. For each target node, the distance between anchor nodes in its range is calculated by using equation ${\hat{d}}_{i}=d_{i}+n_{i}$, where $n_{i}$ is an additive Gaussian noise, and $d_{i}$ is the actual distance that is measured by using the Equation (1):

(1) $$d_{i}=\sqrt{{\left(x-x_{i}\right)}^{2}+{\left(y-y_{i}\right)}^{2}},$$

where the position of target node is denoted as (x,y), and the position of the anchor node is represented as $\left(x_{i},y_{i}\right)$.

The variance of $n_{i}$, as the noise that affects the measured distance between target and anchor nodes is given in Equation (2):

(2) $$\sigma_{d}^{2}=\beta^{2}\cdot P_{n}\cdot d_{i},$$

where $P_{n}$ is the percentage noise in distance measurement $d_{i}\pm d_{i}\left(\frac{P_{n}}{100}\right)$, and β is a parameter whose value is usually set to 0.1 in practical implementations.

**Figure 1:**
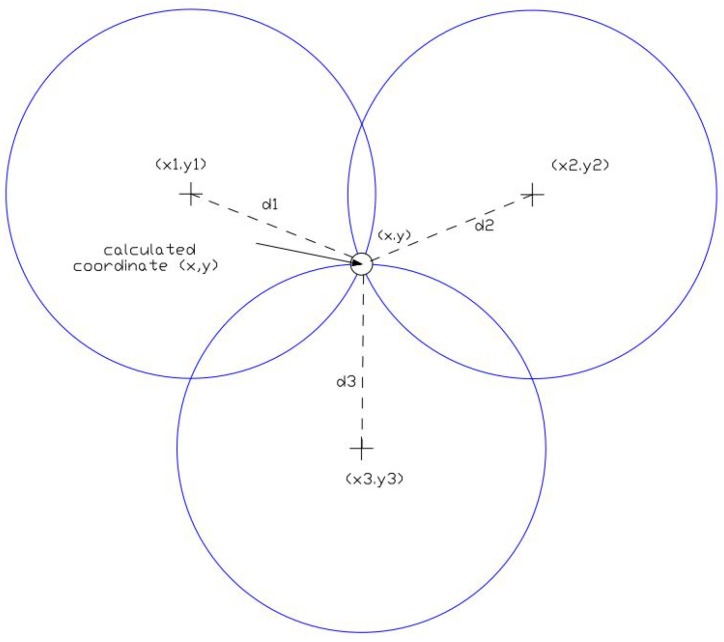
Trilateral positioning method.

In the trilateration method for estimating the position of the unknown sensor node, the target node is defined as localized if there are minimum three anchor nodes with the known coordinates *A*$\left(x_{a},y_{a}\right)$, *B*$\left(x_{b},y_{b}\right)$, and *C*$\left(x_{c},y_{c}\right)$, within its transmission range *R*, and with distance $d_{i}$ from the target node *n*. By utilizing the trigonometric principles of sines and cosines, the position of the target node can be calculated. This minimizes the error between actual distance and the estimated distance. The trilateration method is depicted in Figure 1.

The swarm intelligence metaheuristics which are implemented in this research are executed independently for each localizable target node to obtain its position. The agents are initialized within centroid of the anchor nodes that are located within the range of the localizable target node by using the following expression:

(3) $$\left(x_{c},y_{c}\right)=\left(\frac{1}{N}\sum_{i=1}^{N}x_{i},\frac{1}{N}\sum_{i=1}^{N}y_{i}\right),$$

where *N* presents the total number of anchor nodes within transmission range of target node that is subject to localization.

Furthermore, the swarm intelligence algorithms aim to find the coordinates (x,y) of the target sensor node with the goal to minimize the localization error. The objective function f(x,y) of node localization problem is formulated as the mean square distance between the target node and the anchor node. Mathematical formulation of the objective function is given in Equation (4).

(4) $$f\left(x,y\right)=\frac{1}{N}{\left(\sum_{i=1}^{N}\sqrt{{\left(x-x_{i}\right)}^{2}+{\left(y-y_{i}\right)}^{2}}\right)}^{2},$$

where N≥3 (the trilateration principle requires at least three anchor nodes within transmission range *R* of the localazable target node).

Localization error $E_{L}$ is determined after finding the positions of all target nodes $N_{L}$. It is calculated as the mean of square of the distance between the estimated node coordinates $\left(X_{i},Y_{i}\right)$ and the real node coordinates $\left(x_{i},y_{i}\right)$ which is presented in the Equation (5):

(5) $$E_{L}=\frac{1}{N_{L}}\sum_{i=1}^{N}\sqrt{{\left(x_{i}-X_{i}\right)}^{2}+{\left(y_{i}-Y_{i}\right)}^{2}}$$

The efficiency of the localization algorithm is measured by the average localization error $E_{L}$ and the number of sensor nodes which have not been localized $N_{NL}$, where $N_{NL}=M-N_{L}$. The lower the values of $E_{L}$ and $N_{NL}$, the better the performance and efficiency of the algorithm.

## 4. Original and Dynamic Search Tree Growth Algorithm

As stated in Section 1, the basic focus of the research that is presented in this paper is achieving further improvements in solving the WSNs localization problem by applying swarm algorithms. As the first step in accomplishing this goal, we improved original TGA swarm algorithm.

In our TGA’s implementation, the balance between exploration and exploitation is dynamically adjusted during the algorithm’s execution. Our proposed approach is named dynamic search TGA (dynsTGA).

In this section, we present both the original (that was devised and implemented before our research) and improved version of the TGA, along with the critical analysis and performance discussion.

The TGA algorithm, created by Armin Cheraghalipour and Mostafa Hajiaghaei-Keshteli in 2017 [54], was inspired by the competition among the trees for absorbing food resources. TGA has diverse implementations in vast domains such as global optimization benchmark, engineering constrained benchmarks [55].

At the starting point of the TGA algorithm’s execution, the entire population of solutions is separated into three populations (subpopulations), defined as $N_{1}$, $N_{2}$, and $N_{3}$. Each iteration of the algorithm’s run is divided into four phases. The first phase indicates that the $N_{1}$ better solutions are chosen from the population for conducting the local search procedure. In the second phase $N_{2}$ solutions are moved to the distance between the close best solutions under different α angles. $N_{3}$ worst solutions are rejected from the population in the third phase and replaced by random generated solution from the suitable domain of the search space. Fourth phase is executed by creating $N_{4}$ new randomly distributed solutions. Each solution that is distributed is altered by implementing the mask operator to the best solutions in the population $N_{1}$ that represents the collection of the best solutions, respectively.

The TGA algorithm is executed in the following order. First, the initial population of *N* solutions that are randomly allocated is generated amongst upper and lower paramaters’ bounds, with the fitness value that is calculated for each individual. After this, the entire population of *N* solutions is arranged with respect to the fitness value, where the current best solution $T_{GB}^{j}$ in the *j*-th iteration is defined. The local search for all the individuals in the subpopulation $N_{1}$ is executed by using the Equation (6), where its adapted for each parameter of solution *i*:

(6) $$T_{i}^{j+1}=\frac{T_{i}^{j}}{\theta }+rT_{i}^{j},$$

where $T_{i}^{j}$ and $T_{i}^{j+1}$ present the *i*-th solution in the subpopulation $N_{1}$ in the iteration *j* (old solution *i*) and j+1 (new solution *i*), respectively, θ represents the reproduction rate parameter, and r∈[0,1] is pseudo-random number.

When the new solution is created, the greedy selection is conducted between the old and the new solution, where all the solutions which values are lower are rejected from the population.

Afterwards all the solution from the $N_{2}$ subpopulation are guided towards the two proximate solutions from the $N_{1}$ population under divergent α angles. In order to obtain the closest solutions Equation (7) is considered to all solutions’ parameters.

(7) $$d_{i}=\sqrt{\sum_{i=1}^{N_{1}}{\left(T_{N_{2}}^{j}-T_{i}^{j}\right)}^{2}},where\;\;d_{i}=\left\{\begin{array}{c} d_{i},\;\;if\;\;\;T_{N_{2}}^{j}\neq T_{i}^{j}\\ \infty ,\;\;if\;\;\;T_{N_{2}}^{j}=T_{i}^{j} \end{array}\right.$$

When distance $d_{i}$ is calculated, solutions $x_{1}$ and $x_{2}$ which have the minimal distance from each solution $T_{N_{2}}^{j}$ are segregated for creating linear combination by utilizing the Equation (8):

(8) $$y=\lambda x_{1}+\left(1-\lambda \right)x_{2},$$

where λ∈[0,1] presents the control parameter.

All the solutions $T_{N_{2}}^{j}$ from $N_{2}$ subpopulation are conveyed in the direction between the two adjacent solutions with different α angles according to Equation (9).

(9) $$T_{N_{2}}^{j}=T_{N_{2}}^{j}+\alpha_{i}y$$

When it comes to the subpopulation $N_{3}$, the worst populations are discarded from the population. They are replaced with evenly solutions generated from the feasible domain of the search space. Then, the new population is generated *N*, where it combines $N=N_{1}+N_{2}+N_{3}$. The relatively new $N_{4}$ subpopulation which consists of random solutions created and modified by mask operator in respect to the best solution in subpopulation $N_{1}$. When mask operator is implemented, all the solutions from the $N_{4}$ subpopulation are joined with the population *N*.

The created extended population $N+N_{4}$ is defined by the fitness, where the best *N* solutions are pointed and chosen as the initial population for the next iteration of the TGA algorithm’s run. For this process the roulette wheel selection process is employed.

### Dynamic Search TGA Metaheuristics

Every swarm intelligence metaheuristics performs the search guided by two basic processes: the exploitation (intensification) and exploration (diversification). The algorithm will obtain satisfying results only if the trade-off between these two process is well adjusted.

If the balance between diversification and intensification is not properly adjusted, the algorithm may fail to obtain satisfying results according to two different scenarios. In the first case, if the balance is set in favor to exploitation, the metaheuristics may converge to the sub-optimal part of the search space in early iterations. As a consequence, the search process may be trapped in the local optimum. This behavior is in the literature known as the premature convergence. In the second scenario, if the exploitation exploration balance is adjusted in favor to exploration, in most cases, the algorithm will find the part of the search region where an optimal or satisfying solutions reside, but will fail to hit those solutions due to the deficiency in exploitation power.

In the basic implementation of the TGA approach, the trade-off between intensification and diversification is adjusted by using two control parameters θ and λ, and the number of solutions in the subpopulation $N_{3}$. Recall from Section 4, the subpopulation $N_{3}$ is comprised of the worst solutions that are discarded from the population in each algorithm’s iteration.

Both parameters, θ and λ, control the process of exploitation. Parameter θ directs the local search process, while the basic function of the λ parameter is to direct solutions towards better solutions in the population. On the other hand, with the number of solutions in $N_{3}$ subpopulation, the process of exploration is adjusted.

In the original TGA implementation, all three parameters are static during the whole course of algorithm’s execution.

By performing experimental tests on standard unconstrained benchmarks, we came to the conclusion that the performance in terms of convergence speed and the solution’s quality of the original TGA approach can be improved by introducing dynamical adjustments of the values for θ and $N_{3}$ control parameters.

In the early iterations of algorithm’s execution, the value of the parameter θ should be lower, and the novel solution should not be too close to the current solution (Equation (6)). The basic assumption behind this theory is that in the beginning of execution, the algorithm did not hit the right part of the search space, hence the exploitation should be decreased. In later iterations, the value of the parameter θ should be higher with the assumption that the algorithm has found the part of the search space where an optimum solution resides.

Also, in the beginning of the algorithm’s execution, the number of solutions that are removed from the population (subpopulation $N_{3}$) should be higher, because in this phase more exploration power is needed. Since the change of the $N_{3}$ value has direct implications to the values of $N_{1}$, and $N_{2}$, these parameters should be adjusted as well.

Thus, during the course of the algorithm’s execution, the value of θ is gradually increased, while the number of solutions in the subpopulation $N_{3}$ is gradually decreased.

By conducting experimental simulations, we concluded that value of the λ parameter should be static during the whole course of algorithm’s execution (Equation (8)). We came to this conclusion in a “trial and error” manner, by conducting experiments with dynamic λ adjustments. On average, when the λ was dynamically adjusted, the results were worse than in the cases with static λ value.

With the introduction of dynamic behavior of θ and $N_{3}$ into basic TGA implementation, we devised improved dynsTGA metaheuristics. The dynsTGA pseudo-code is presented in Algorithm 1.

**Algorithm 1** Pseudo code of the dynsTGA metaheuristics **Initialization**. Generate random initial population, set the iteration counter *t* to 1, the value of maximum iteration number (MaxIter), and the initial values of θ and $N_{3}$ control parameters **while**
t<MaxGen
**do**  Evaluate all solutions in the population and sort them according to their fitness value  **for all** solutions in $N_{1}$
**do**   Conduct local search by utilizing Equation (6)   Apply greedy selection mechanism to choose between the old and the new solution  **end for**  **for all** solutions in $N_{2}$
**do**   Move solutions towards the closest best solutions in $N_{1}$ by using Equations (7)–(9)   Apply greedy selection mechanism to choose between the old and the new solution  **end for**  Remove $N_{3}$ worst solutions from the population and replace them with randomly solutions from the search domain  Generate $N_{4}$ randomly distributed solutions and modify each solution in respect to the best solutions in $N_{1}$ by using the mask operator  Evaluate all solutions in the population and sort them according to their fitness value  Choose *N* best solutions as initial population for the next iteration  Recalculate the values of θ and $N_{3}$ along with $N_{2}$ and $N_{1}$ control parameters **end while** **return** The best solution from the population

## 5. Original and Hybridized Elephant Herding Optimization Metaheuristics

Similarly, as in the case of the TGA metaheuristics, as the second step to achieve the goal of making further improvements in solving the WSNs localization problem by applying swarm algorithms, we improved original EHO metaheuristics by hybridization.

In this section, we present both the original (that was devised and implemented before our research) and hybridized version of the EHO, along with the critical analysis and performance discussion.

Elephant herding optimization (EHO) algorithm, proposed and created by Wang et al. in 2015 for solving global optimization tasks [56], was inspired by the social communication and behavior of elephants in herds. EHO has many implementation in different domains, such as node localization in WSNs [4], standard benchmark problems [57], static drone placement problem [58], multilevel image threshold [59], etc. The general-purpose heuristic search is designed to indicate the social coexistence of the elephants in herds under the leadership of a matriarch, whence fully grown male calves leave the clan to live independently, but still communicate with the rest of the herd.

Because of this independence and structural communication between elephants in the same clan, and outside of the clan, there are two different environments: the first environment where elephants communicate and function under the influence of a matriarch, and the second environment where male calves leave the herd. These environments are defined as updating and separating operators.

In the population, each individual is presented as an integer number vector with the dimension 2N, where *N* indicates the number of sensor nodes randomly distributed in the monitoring field. Each population is detached into *n* clans.

Then each solution *j* in each clan ci in the EHO algorithm is updated by its present position and matriarch ci through an updating operator. Afterwards, the population dissimilarity is enhanced through the separating operator at the next generations of the algorithm’s execution.

The population is divided into *n* clans. The updating operator is defined by changing the position of each solution *j* in the clan ci by the impact of the matriarch ci which has the best fitness value in generation which can be seen in Equation (10):

(10) $$x_{new,ci,j}=x_{ci,j}+\alpha \times \left(x_{best,ci}-x_{ci,j}\right)\times r,$$

where $x_{new,ci,j}$ is the new position of the individual *j* in the clan ci, and $x_{best,ci}$ is the best solution in the clan ci found at this time, where $x_{ci,j}$ represents the old position of the individual *j* in the clan ci. Parameter α∈[0,1] is a scale indicator that designates the authority of the matriarch ci on $x_{ci,j}$, and r∈[0,1] is a random variable with uniform distribution. To update the fittest solution in each clan ci:

(11) $$x_{new,ci,j}=\beta \times x_{center,ci},$$

where β∈[0,1] presents the factor that impact the $x_{center,ci}$ on the updated individual.

As *D* indicates the total dimension of the search space, following is the calculation of the center of the clan ci, $x_{center,ci,d}$ for d−th dimension problem:

(12) $$x_{center,ci,d}=\frac{1}{n_{c}i}\times \sum_{j=1}^{d}x_{ci,j,d},$$

where $1<x_{center,ci}<d$, $n_{c}i$ is the number of elephants in clan ci, and $x_{c}i,j,d$ is the *d*-th of the elephant individual $x_{c}i,j$.

The separating operator can be modeled as:

(13) $$x_{worst,ci}=x_{min}+\left(x_{max}-x_{min}+1\right)\times rand,$$

where $x_{max}$ and $x_{min}$ are upper and lower bound of the position of the individual, respectively. $x_{worst,ci}$ is the worst individual in clan ci, and rand∈[0,1] is a random number chosen by uniform distribution.

### Hybridized Elephant Herding Optimization

By conducting empirical tests on standard unconstrained and constrained benchmarks of the basic EHO metaheuristics, we have noticed some deficiencies in the algorithms’ performance.

As already mentioned above, robustness and solution’s quality of any swarm intelligence approach depends on the balance between the exploitation and the exploration. In the original EHO approach, the process of intensification is performed by the updating operator, and the new position (solution) depends of the current best solution in the population (see Equations (10)–(12)). The process of diversification is modeled by separating operator (Equation (13)).

According to the performed practical tests on standard unconstrained and constrained benchmarks, we concluded that the basic EHO metaheuristics suffers from inappropriate trade off between exploitation and exploration. Utilization of the separating operator, which performs exploration, is not enough, and this balance is shifted towards exploitation. As a consequence of inadequately established balance, in some runs, the algorithm could not hit region of the search space where an optimal solution resides, and it gets stuck in a suboptimal domain. Such behavior has direct implications to the worse mean values of the objective function that is subject to the optimization process. On the other hand, in some runs, the original EHO is lucky, and manages to find the right part of the search space. Thus, we concluded that the basic EHO has two drawbacks: the lack of exploration power and inadequate balance between exploration and exploitation.

To overcome these deficiencies, we hybridized EHO with the well-known ABC swarm intelligence metaheuristics [60]. We introduced another criteria for discarding solutions from the population—the number of cycles (iterations) in which a particular solution cannot be improved by the process of exploitation. For this purpose, we adopted a limit parameter from the ABC metaheuristics, similar to Ref. [61].

For describing solution in the population, in our hybridized approach we used limit as an additional attribute. In each iteration of algorithm’s execution, when a solution cannot be improved, the value of its limit parameter is incremented by one. When a value of the limit for a particular solution reaches a threshold (th) value, the solution is discarded from the population and replaced with the pseudo-random solution. The replacing solution is generated by using Equation (13).

By incorporating the limit parameter from the ABC approach, hybridized EHO obtains better intensification-diversification balance by increasing the intensity of exploration.

At the beginning of algorithm’s execution, since for modeling exploration two mechanisms are used, the trade off of exploitation-exploration is slightly more shifted towards exploration. The basic assumption is that at these early iterations, the algorithm still did not find the right part of the search space, and more exploration is needed. However, in later iterations, with the preposition that the algorithm has converged to an optimal region, more intensive exploitation is needed and the balance should be shifted towards exploitation. In order to accomplish such behavior, the value of the limit parameter in early iterations (cycles) is is lower, and then as the execution approaches the termination condition (the maximum number of iterations), the value of the limit is gradually increasing. In this way, in later iterations, the solutions are rarely discarded from the population and the balance between exploitation and exploration is shifted towards exploitation.

We named our approach hybridized EHO (HEHO). The pseudo-code is given in Algorithm 2.

**Algorithm 2** Pseudo code of the HEHO algorithm **Initialization**. Generate the individuals in population; divide population into *n* clans; calculate fitness for each individual; set generation counter t=1, maximum generation MaxGen, and the initial value of the limit parameter **while**
t<MaxGen
**do**  Arrange all solutions according to their fitness  **for all** clans ci
**do**   **for all** solution *j* in the clan ci
**do**    Update $x_{ci,j}$ and generate $x_{new,ci,j}$ using Equation (10)    Select and obtain better solution between $x_{ci,j}$ and $x_{new,ci,j}$    Update $x_{best,ci}$ and generate $x_{new,ci,j}$ using Equation (11)    Select and retain better solution between $x_{best,ci}$ and $x_{new,ci,j}$   **end for**  **end for**  **for all** clans ci in the population **do**   Replace the worst solution in clan ci using Equation (13)   Replace the solutions that violate limit value by using Equation (13)  **end for**  Analyze population and calculate fitness  Recalculate the value of the limit parameter **end while** **return** the best solution among all clans

## 6. Empirical Results and Analysis

The experimental section of this paper is divided into two parts. In the first part, since the WSNs localization problem can be formulated as bound-constrained optimization, we show testing results and analysis of the dynsTGA and HEHO metaheuristics for standard bound-constrained (unconstrained) optimization benchmarks. In the second part we show empirical results for the WSNs localization problem along with comparative analysis with other state-of-the-art metaheuristics, theoretical analysis, and discussion.

### 6.1. Testing Results for Unconstrained Benchmarks

As already mentioned in Section 4 and Section 5, we first tested devised approaches dynsTGA and HEHO on standard unconstrained (bound constrained) benchmarks. We wanted to measure improvements in terms of robustness and solutions’ quality of our modified versions over basic implementations of the TGA and EHO metaheuristics.

Since the WSN localization problem belongs to the group of NP hard problems, if devised approaches perform better than the original ones in the case of unconstrained benchmarks, the logical assumption is that they will also obtain better results in the case of WSNs localization.

Details of some benchmark functions that were used for validation of our approaches are given in Table 1.

All tests for both algorithms are executed in 30 independent algorithm runs, each starting with different pseudo-random number seed.

#### 6.1.1. TGA and dynsTGA Control Parameter Setup and Testing Results

For evaluating performance of the TGA and dynsTGA we employed similar control parameter settings as in Refs. [54,55]. The initial population size (*N*) was set to 100, and the values for $N_{1}$ and $N_{2}$ were set to 20. The value for $N_{3}$ was set to 60 ($N_{3}=N-(N_{1}+N_{2}$). The value of $N_{4}$ was set to 40, while the maximum iteration number (MaxIter) was set to 250. The other control parameters were adjusted as follows: λ=0.5 and θ=0.2. This yields to the total number of 35,000 function evaluations.

The dynsTGA approach utilized similar values to the control parameters described above, with few modifications. In the first 210 iterations, the values of $N_{1}$, $N_{2}$, and $N_{3}$ were static. After, in every of the iterations between 210 (inclusive) and 250 (inclusive), the value of $N_{3}$ was decremented by one, and the values of $N_{1}$ and $N_{2}$ were incremented by 1 in even and odd iterations, respectively. The value for control parameter $N_{4}$ was static during the whole course of algorithm’s execution. The initial value of the θ parameter was set to 0.2. However, the θ value was adjusted in each iteration according the expression $\theta^{j+1}=\theta^{j}\cdot 1.002$, until the threshold value 1.5 is reached.

Comparative analysis between the original TGA and dynsTGA for unconstrained tests is given in Table 2. As performance indicators, we used best (best solution obtained in a set of 30 independent algorithm runs), mean (the average of all bests obtained in each of 30 runs), and standard deviation (standard deviation of all bests obtained in each of 30 runs). In Table 2, the best results obtained for each performance indicator are marked bold.

As can be seen from Table 2, in the case of most tests for all three indicators, the dynsTGA significantly outperformed the original TGA implementation.

#### 6.1.2. EHO and HEHO Control Parameter Setup and Testing Results

Both approaches EHO and HEHO were adjusted as follows: the number of clans *n* was set to 5, and the number of solutions in each clan $n_{ci}$ was set to 10, similarly to Refs. [56,57]. Each run of algorithms was executed in 700 iterations, yielding a total number of 35,000 objective function evaluations. The values for the scale factors α and β were set to 0.5 and 0.1, respectively.

In the case of the HEHO approach, the initial value of the limit parameter was set to 5. Then, in each even iteration, the limit value was incremented by one. In this way, in early iterations, the solutions that cannot be improved were discarded from the population often and exploration power is higher. However, in later iterations with the increase of the limit parameter value, solutions were discarded from the population with lesser frequency, and the trade-off between exploitation and exploration is shifted in favor of exploitation.

Comparative analysis between the original EHO and the HEHO for standard bound-constrained benchmarks is given in Table 3. Similarly, as in the case of Table 2, the best results for each category of performance indicators are marked bold in Table 3.

From Table 3 it can be concluded that in all tests, except for the benchmark F8, the HEHO obtains better performance than the original EHO approach. Also, if we compare results of the dynsTGA and HEHO (Table 2 vs. Table 3), it can be seen that the dynsTGA performs slightly better in most benchmark cases. Since the WSN localization problem is categorized as NP hard with bound constraints (like presented benchmark functions), in this phase of the research we expected that the dynsTGA will also outperform the HEHO approach when tackling this problem.

#### 6.1.3. Comparative Analysis with Other Metaheuristics

In the final part of testing dynsTGA and HEHO metaheuristics on standard bound-constrained benchmarks, we performed comparative analysis with other state-of-the-art algorithms that have been tested on the same problem instances. We also wanted to perform side-by-side comparison between the dynsTGA and HEHO approaches, so we included results for both proposed approaches into single comparison table.

Comparative analysis was performed with the iterative best performance algorithm (IBPA), largest absolute difference algorithm (LADA), taboo search (TS), and weighted superposition attraction (WSA) metaheuristics. Simulation results for the algorithms that are included in comparative analysis were taken from Refs. [55,62].

Comparative analysis is shown in Table 4. The best results for each performance metrics are marked bold.

From the presented comparative analysis it can be concluded that on average the dynsTGA metaheuristics performs better than all other approaches included in comparisons. For example, in the case of the F3 benchmark, the dynsTGA obtained better mean and standard deviation results than all other approaches, while both proposed metaheuristics, dynsTGA and HEHO, managed to accomplish optimal solutions. Also, in the F6 test, the dynsTGA showed the best performance for best, as well as for the mean indicator, while the HEHO obtained the best value for the standard deviation metric.

Moreover, as it has already been pointed out in Section 6.1.2, when performing comparison between the dynsTGA and the HEHO, it can be seen that in most benchmark cases, the dynsTGA performs slightly better, and at this point of conducted research we expected that the dynsTGA will also outperform the HEHO approach when tackling the WSNs localization challenge.

**Figure 2:**
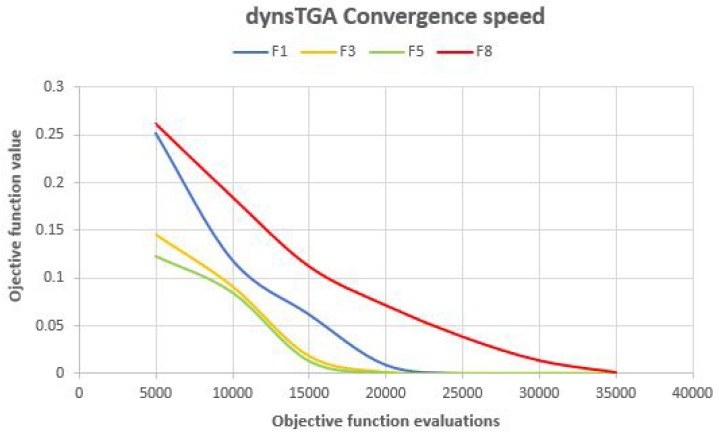
Dynamic search tree growth algorithm (dynsTGA) convergence speed for some unconstrained benchmarks.

**Figure 3:**
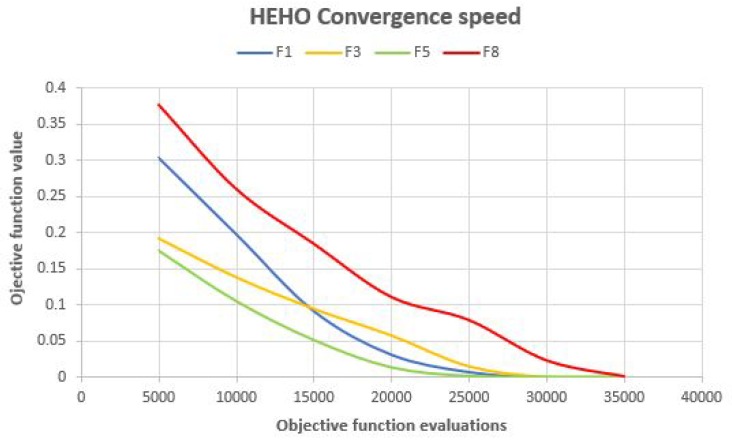
Hybridized elephant herding optimization (HEHO) convergence speed for some unconstrained benchmarks.

In order to make better insights into the proposed algorithms’ performance, convergence speed graphs for F1, F3, F5, and F8 benchmarks for the dynsTGA the HEHO are shown in Figure 2 and Figure 3, respectively. From the presented figures, it can be clearly seen that the dynsTGA metaheuristics achieves better convergence speed and converges faster to the global optimum. This further means that the dynsTGA has better exploitation (intensification) ability than the HEHO. Also, at some points of execution, the HEHO gets trapped in suboptimal domains of the search space. For example, in simulations with F1, F3, and F5 benchmarks, the dynsTGA obtained its global optimum after 20,000 objective function evaluations, while it took around 25,000 objective function evaluations for the HEHO to achieve its global optimum in the same tests.

### 6.2. WSN Localization Problem

In this subsection, we first present the setup of experimental environment for the WSN localization problem and the control parameters’ adjustments for EHO, HEHO, TGA, and dynsTGA metaheuristics that were employed in the conducted experiments. After we show details of conducted simulations, as well as comparative analysis with other state-of-the-art approaches found in the literature that were tested on the same problem instance under the same experimental conditions.

#### 6.2.1. Experimental Setup and Parameter Adjustments

In this and the subsequent subsection, we have provided exhaustive details of the control parameters’ adjustments and experimental conditions, so researchers who wants to implement the proposed approaches and to run simulations have more than enough information to do this on their own.

For the experimental setup of this paper we have constructed a simulation topology which includes a two-dimensional (2D) WSN monitoring environment (deployment area) with a size of 100U×100U, where *U* represents unit of measurement. Within this monitoring environment, static target sensor nodes and anchor nodes with coordinates (x,y) are randomly deployed between the lower and the upper boundary of the WSN monitoring domain, using pseudo-random number generator.

We conducted experiments with different number of target nodes *M* and anchors nodes *N*. We wanted to see how algorithms behave in different test scenarios. In performed all simulations, the number of target nodes varies in the range [25,150], while the number of anchor nodes in all simulations is between 8 and 35 (inclusive). In every run of the algorithm, the network topology was generated randomly. Similar experiments were conducted in Ref. [15].

As explained in Section 3, the range measurement is blurred with the additive Gaussian noise ${\hat{d}}_{i}=d_{i}+n_{i}$. The parameter $\sigma_{d}$, which represents the standard deviation of the measured distance, influences the performance of localization (see Equation (2)). Two essential parameters that affect the localization error $E_{l}$ are density of anchor nodes per $U^{2}$ and sensors’ transmission range *R*. The transmission range is set to 30U in all simulations.

Also, in all conducted experiments with all algorithms (TGA, dynsTGA, EHO, and HEHO), the size of population *N* and the maximum generation number (MaxGen) were set to 30 and 200, respectively. The same parameter adjustments were used in Ref. [15]. In Ref. [15], swarm intelligence approaches that are used for the purpose of comparative analysis in this paper are presented. By utilizing this set of parameters, a comparative analysis presented in this study is more objective and represents real performance comparison between different swarm algorithms approaches.

The basic TGA and dynsTGA control parameters were adjusted as follows: initial population size (*N*) was set to 25, the values for $N_{1}$ and $N_{2}$ were set to 6, while the value for $N_{3}$ was adjusted to 13. Also, in every iteration, additional 5 solutions were evaluated ($N_{4}=5$). This in total yields 30 solutions in the population, like in Ref. [15]. For other control parameters we set the following values: λ=0.5 and θ=0.2.

In the case of dynsTGA experiments, the value of $N_{4}$ was fixed during the whole course of algorithm’s execution. Also, the values for $N_{1}$, $N_{2}$, and $N_{3}$ were static in the first 180 iterations. In the final stages of algorithm’s execution, the value of $N_{3}$ was decremented by one until a threshold value of $N_{3}=1$ is reached, and the values of $N_{1}$ and $N_{2}$ were incremented by 1 in even and odd iterations, respectively. Finally, in the latest iterations, parameters $N_{1}$ and $N_{2}$ reached the value of 12.

At the beginning of the dynsTGA execution, the value of θ was set to 0.2. In every iteration, setting for the θ was adjusted by utilizing expression $\theta^{j+1}=\theta^{j}\cdot 1.002$, until the threshold value 1.5 is reached. Similar was performed in dynsTGA’s tests for bound-constrained benchmarks (see Section 6.1.1 ).

The basic EHO and HEHO control parameters were set as follows: the number of clans *n* was set to 5, and the number of solutions in each clan $n_{ci}$ was set to 6. The values for the scale factors α and β were set to 0.5 and 0.1, respectively.

The adjustments for the limit parameter in the case of HEHO approach were the same as in unconstrained tests (see Section 6.1.2). The initial value of the limit parameter was set to 5. Then, in each even iteration, the limit value was incremented by 1. In this way, in early iterations, the solutions that cannot be improved were discarded from the population often and exploration power is higher. However, in later iterations with the increase of the limit parameter value, solutions were discarded from the population with lesser frequency, and the trade-off between exploitation and exploration is shifted in favor to exploitation.

In all conducted simulations presented in Section 6.2.2 we utilized Intel CoreTM i7-4770K processor @4GHz with 32GB of RAM memory, Windows 10 Professional x64 operating system, and Java Development Kit 11 (JDK 11) and IntelliJ integrated development environment (IDE).

#### 6.2.2. Empirical Results, Comparative Analysis, and Discussion

In the research conducted for the purpose of this paper we performed two sets of experiments in order to measure real performance of our proposed approaches.

In the first set of experiments, we wanted to measure the influence of the noise percentage ($P_{n}$) in distance measurement on the localization accuracy. For this purpose we ran original and upgraded/hybridized TGA and EHO metaheuristics with the value of $P_{n}$ set to 2 and 5, respectively. In this case we used 40 target nodes (M=40), and 8 anchor nodes (N=8) randomly deployed in the WSN domain.

With each particular value of $P_{n}$ we executed all algorithms in 30 independent runs, and for each run we used different pseudo-random number seed. As performance indicators, we took the following metrics: the mean number of non-localized nodes ($N_{NL}$) and the mean localization error ($E_{L}$). Values of performance indicators were averaged over 30 runs.

The same parameter setup, as well as the performance indicators were used in Ref. [15].

Experimental results are summarized in Table 5. Best results from each category (mean $N_{NL}$, mean $E_{L}$, computation time) are marked bold.

From the results presented in Table 5, it can be noticed that the influence of the percentage noise ($P_{n}$) on localization accuracy is obvious and significant. When the $P_{n}$ was decreased from 5 to 2, in all test case for all approaches, the localization error decreased, while the number of localized nodes increased (number of non-localized nodes decreased).

From the results presented in Table 5 it can be concluded that the dynsTGA metaheuristics obtains the best results. This was expected since dynsTGA also performed the best in the case of conducted tests on standard bound-constrained benchmarks (refer to Table 2 and Table 3). The dynsTGA outperformed other approaches in both performance indicators: it managed to localize most target nodes with low localization error.

Basic TGA and HEHO performed similarly. For example, in the case of the test with $P_{n}$ set to 5, the HEHO localized more targets, but TGA obtained lower localization error. Also, in the case of $P_{n}=2$ test, the difference between these two approaches is insignificant. Finally, original EHO metaheuristics was significantly worse than all other approaches presented in this paper.

When analyzing computational costs in terms of execution speed, it is obvious that the dynsTGA is the most expensive approach, while the basic TGA implementation proved to be light-weight and executed faster than other metaheuristics. These results are expected since the dynsTGA at the end of each iteration performs dynamical adjustment of the control parameters $N_{1}$,$N_{2}$, $N_{3}$, and θ.

In Ref. [15], the same tests were performed (with $P_{n}=2$ and $P_{n}=5$) under the same experimental conditions and under the same problem instance. In this paper, the authors proposed butterfly optimization algorithm (BOA) and compared it with the particle swarm optimization (PSO) and the firefly algorithm (FA).

We wanted to see how approaches presented in this paper perform related to approaches showed in Ref. [15], and for this purpose we conducted comparative analysis. A side by side comparison is presented in Table 5 and Table 6.

The comparison could be made only for the mean number of non-localized nodes ($N_{NL}$) and the mean localization error ($E_{L}$) metrics. Comparative analysis for the computational time indicator could not be performed, since the approaches presented in Ref. [15] were tested on different computational platforms. In the presented table, the best results from each category are marked bold.

According to the results presented in Table 6, a general conclusion is that the dynsTGA proved to be the best approach. In both cases, for $P_{n}=2$ and $P_{n}=5$ the dynsTGA obtained better results than all other metaheuristics included in comparative analysis. The second best approach is BOA. For example, when $P_{n}$ was set to 5, the dynsTGA managed on average to localize 35.5 targets, while BOA successfully localized 35.3 targets. Also, the dynsTGA obtained lower localization error (0.19 vs. 0.28).

Similar results are obtained in the case of $P_{n}=2$ test, where the dynsTGA on average could not localize 4.3 targets, while BOA could not find the position of 4.5 nodes. Also, in this test, the dynsTGA localized targets with a localization error that is almost 24% lower than BOA (0.16 vs. 0.21).

Also, from Table 6 it can be seen that the third best performing algorithm included in comparative analysis are TGA and HEHO. These two approaches share the third place. A more detailed comparison between the TGA and HEHO is given above.

The fourth best performing approach is PSO. In both tests, the PSO proved to be the average performing metaheuristics method for this type of problem. Finally, the last place is shared between the FA and the EHO. It should be also noted that the FA performed slightly better than EHO, but this difference is negligible. For example, in the test with $P_{n}$ set to 2, both algorithms on average managed to localize 33.8 out of 40 targets, where the FA obtained slightly lower localization error (0.69 vs. 0.71).

In the second set of experiments, we utilized different numbers of target nodes (*M*) and sensor nodes (*N*). We wanted to perform more detailed analysis of robustness and solutions’ quality of proposed approaches. In all tests the value of $P_{n}$ was set to 2, and each experiment is conducted in five independent runs.

Other experimental and metaheuristics control parameters were adjusted as shown in Section 6.2.1.

In Table 7, we show detailed results for all five runs in all conducted experiments. The annotations used in the results table are the following: number of localized nodes, localization error, and execution time measured in seconds are represented with $N_{L}$, $E_{L}$, and $T_{E}$, respectively.

Since the proposed localization algorithms are stochastic, different results are obtained in different runs and the best results from each category of tests could not be emphasized.

As can be seen from the presented table, the results vary from run to run. For example, in the test with 25 targets and 10 anchor nodes, the EHO meatheuristics managed to localize between 17 and 21 targets, while the dynsTGA localized 23, 24, or 25 nodes with the unknown position. Also, in this test, the localization error in all runs is uniformly better in the case of dynsTGA metaheuristics, than in the case of other approaches. Metaheuristics HEHO and TGA perform similarly, and the performance difference is negligible, like in the test with 40 target and 8 anchor nodes (please refer to Table 5).

In the experiment with 125 target and 30 anchor nodes, the EHO localized between 121 and 125 targets, and dynsTGA in almost every run managed to determine positions of unknown nodes. The EHO and TGA localized between 123 and 125 targets. Also, in this test in almost all runs, the dynsTGA obtained the lowest localization error.

Regarding the execution speed, the most expensive approach is dynsTGA, while the algorithm that consumes the least amount of computational resources is TGA. Since the HEHO incorporates limit parameter from the ABC metaheuristics, this approach utilizes slightly more resources that its basic implementation.

Similar conclusions can be drawn from all other tests. Considering both performance indicators (number of localized nodes and localization error), the worst performing metaheuristics is EHO, while the algorithm that obtained the best solution quality is the dynsTGA. Metaheuristics HEHO and TGA generate nearly same results in all test instances.

**Figure 4:**
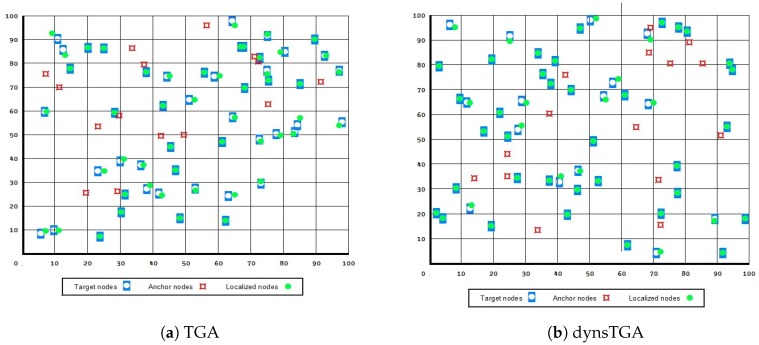
Node localization using original TGA and dynamic TGA.

**Figure 5:**
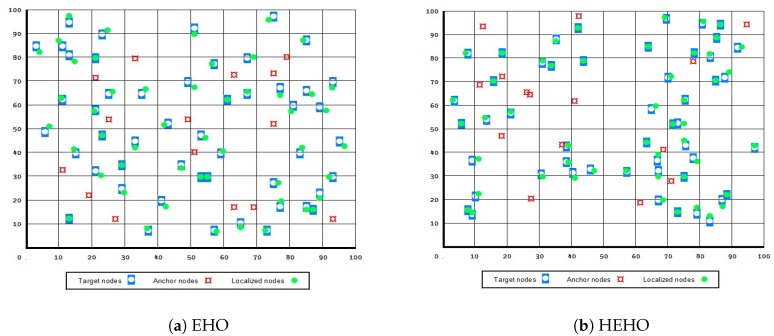
Node localization using original EHO and hybridized EHO.

A visualization of the results in the test with 50 target and 15 anchor nodes, in the case when all presented approaches managed to localize all 50 unknown sensors, is given in Figure 4 and Figure 5. From the presented figures it can be clearly seen that the dynsTGA obtains the lowest localization error, while the basic EHO approach localized all 50 targets with the least precision and accuracy.

As already mentioned above, in Ref. [15] the implementation of BOA for the same problem instance and under the same experimental conditions was presented. Also, in Ref. [15] detailed analysis of results for the five algorithms’ runs was given. In Table 8, we show comparative analysis between our HEHO and dynsTGA implementations and BOA, FA, and PSO showed in Ref. [15].

By carefully analyzing Table 8, it can be seen that dynsTGA performs better than all other approaches included in comparative analysis. The second best metaheuristics is BOA, while TGA and HEHO outperform the FA and PSO algorithms. Similar deduction was drawn in tests with varying $P_{n}$ values (please refer to Table 6).

For example, in tests on problem instances with {50 targets, 15 anchors}, {100 targets, 25 anchors}, and {150 targets and 35 anchors} the BOA approach on average obtained localization errors of 0.30, 0.24, and 0.55, respectively. For the same tests, the dynsTGA-based localization localized unknown targets with the localization errors of 0.25, 0.22, and 0.47, which is significantly better than in the case of BOA. At the same time, on average, both algorithms managed to localize the same number of target nodes.

Contrarily, the HEHO metaheuristics, which outperform the PSO and FA approaches, for the same problem instances, obtained localization error of 0.37, 0.35, and 0.78, respectively. Thus, the HEHO-based localization estimated the locations of unknown targets with lesser accuracy when compared to the dynsTGA approach.

**Figure 6:**
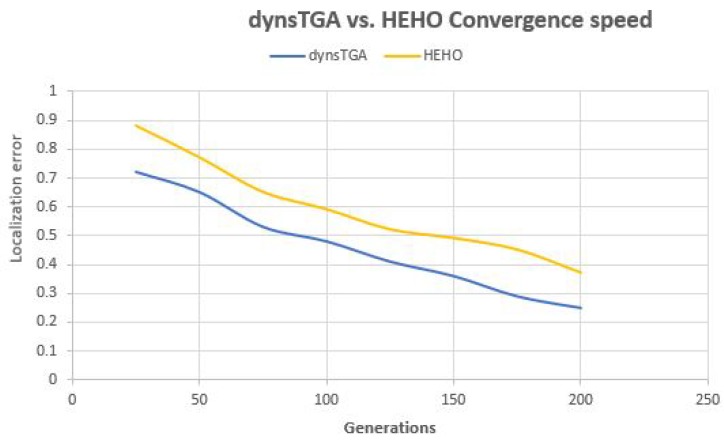
Convergence speed—dynsTGA vs. HEHO (50 targets and 15 anchors).

**Figure 7:**
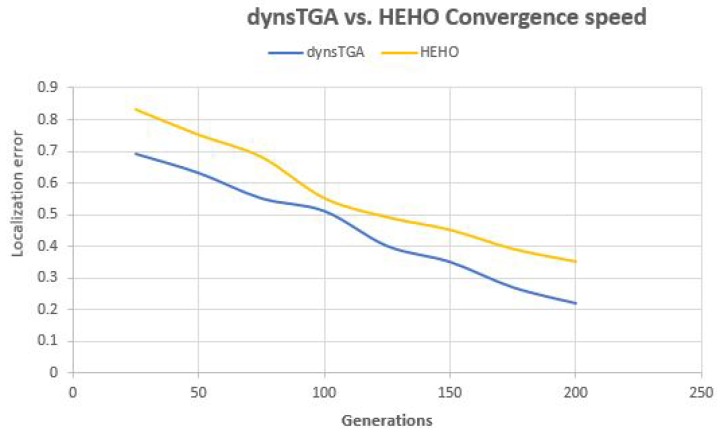
Convergence speed—dynsTGA vs. HEHO (100 targets and 25 anchors).

**Figure 8:**
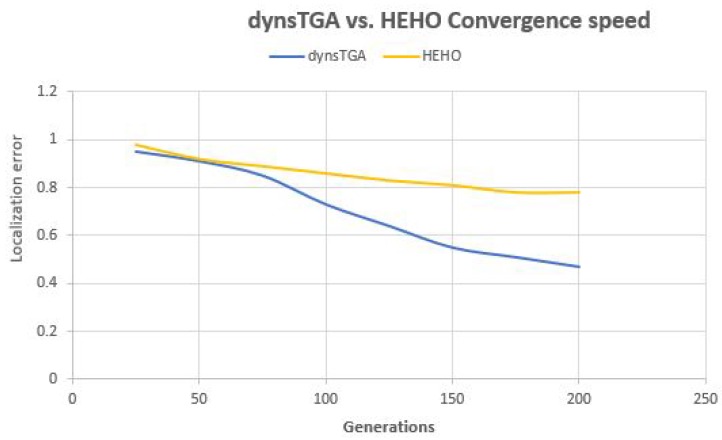
Convergence speed—dynsTGA vs. HEHO (150 targets and 35 anchors).

Side-by-side comparisons of convergence speed between the dynsTGA and the HEHO on problem instances with {50 targets, 15 anchors}, {100 targets, 25 anchors}, and {150 targets and 35 anchors} are shown in Figure 6, Figure 7, and Figure 8, respectively. For visualization purposes, the averaged results over 30 independent algorithms’ runs are taken.

From the presented figures, it can be clearly seen that the dynsTGA obtains much better performance and convergence speed than the HEHO approach. In the case of the problem instance with 150 targets and 35 anchor nodes (Figure 8), in early generations the dynsTGA converges slower to the optimum part of the search space. However, in later generations, the converges speed of the dynsTGA is improved due to the dynamical adjustments of $N_{3}$ and θ control parameters.

#### 6.2.3. Additional Tests

Finally, we have also performed additional experiments with varying transmission range, number of anchor nodes, and number of generations. We wanted to measure the impact of experimental conditions changes on the obtained results and to better establish performances of the proposed metaheuristics. As in the previously conducted experiments, results are averaged over 30 independent algorithms’ runs, each starting with different pseudo-random number seed.

**Figure 9:**
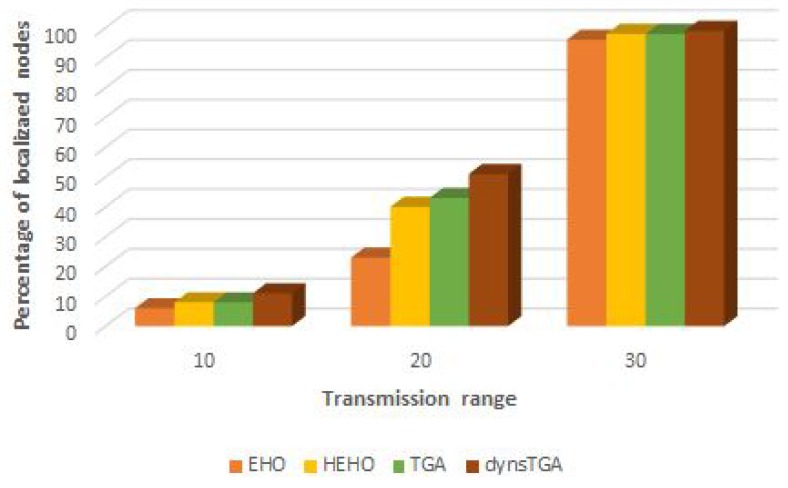
Percentage of localized nodes vs. transmission range.

In the experiments with varying transmission range, we executed algorithms several times with different values for anchor nodes’ transmission range (from 10U to 30U). From the conducted simulations, it is evident that with the increase in the transmission range of the anchor nodes, the number of localized sensors also increases. Visual representation of results for the problem instance with 25 anchor and 100 target nodes is shown in Figure 9.

**Figure 10:**
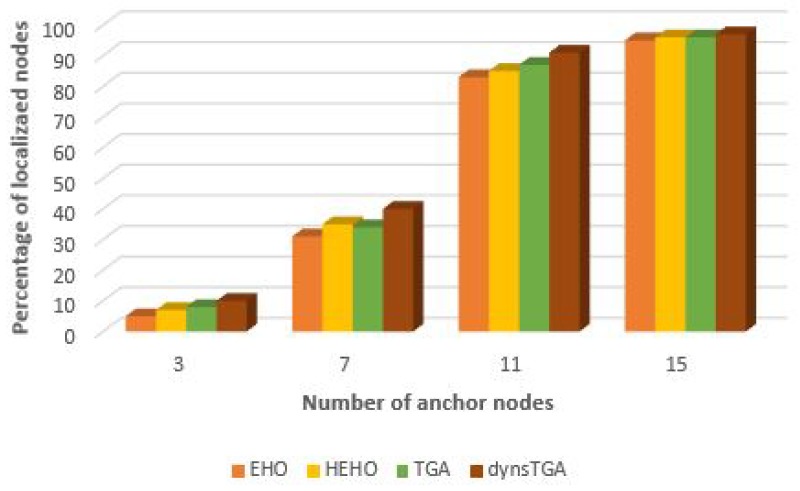
Percentage of localized nodes vs. number of anchor nodes.

With the increase of anchor node density, the location estimation accuracy, as well as the number of localized nodes increase. To prove this, we have executed algorithms several times with varying number of anchor nodes (from 3 to 15) on the problem instance with 50 target nodes. Visual representation of the obtained results (percentage of localized nodes vs. number of anchor nodes) is given in Figure 10.

**Figure 11:**
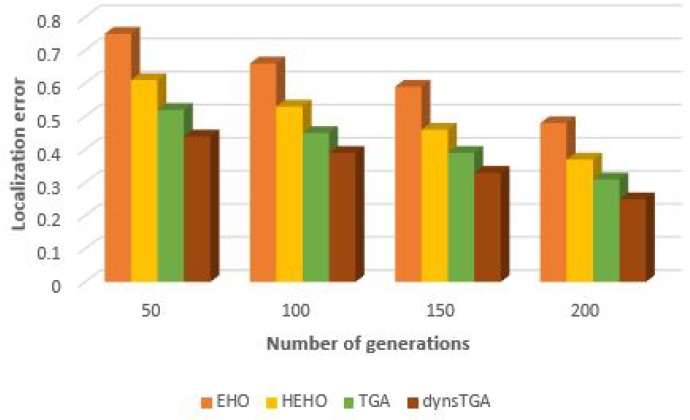
Localization error vs. number of generations.

With the goal to measure the performances of the proposed approaches with greater accuracy, we have also performed experimental simulations with varying number of generations on the WSN topology with 50 target and 15 anchor nodes. With the increase in generation number, the number of localized nodes also increases, and the localization error decreases. The dynsTGA-based localization obtained the least localization error compared to other proposed approaches and proved to be the best metaheuristics when tackling this problem. However, as it can be seen from Table 7, the dynsTGA is the most expensive in terms of execution time and the utilization of computing resources. Visual representation of results (number of generations vs. localization error) is given in Figure 11.

## 7. Conclusions and Future Work

The research presented in this paper is aimed towards achieving further improvements in solving the WSNs localization problem by employing swarm intelligence algorithms. To accomplish this goal, we have improved the basic versions of the TGA and EHO swarm intelligence metaheuristics and applied them in determining the locations of unknown sensor nodes with greater accuracy and precision than other similar algorithms.

The scientific contribution of this paper is twofold: improvements in solving the WSNs localization problem are established and the original versions of the TGA and EHO swarm intelligence metaheuristics are improved.

In the basic TGA, with the goal to enhance exploitation-exploration balance, we introduced dynamic behavior and devised dynsTGA metaheuristics. We have also enhanced the original EHO approach by incorporating the limit control parameter from the ABC swarm algorithm, which controls the process of diversification. We named our approach HEHO.

Both proposed algorithms (dynsTGA and HEHO) were first tested on standard unconstrained (bound-constrained) benchmarks with the objective to measure improvements over the original (unmodified) versions.

With the goal to improve the localization accuracy of unknown targets, we have conducted empirical experiments on different sizes of sensor networks ranging from 25 to 150 target nodes, whose distance measurements are corrupted by Gaussian noise. To establish whether we accomplished our objective, we performed comparative analysis with other state-of-the-art swarm intelligence algorithms, the BOA, PSO, and FA, which have been already tested on the same localization problem instance and under the same experimental conditions. As a general conclusion, the dynsTGA metaheuristics showed the best performance, solution quality, and robustness when tackling the node localization problem and improved results of the previously tested algorithms.

All the details, including descriptions of the algorithms’ implementations, experimental environment setup, and control parameters’ adjustments, are fully provided, so researchers who wants to implement the proposed approaches and to conduct simulations on their own could do so based on the information provided in this paper.

As part of the future research in this domain we will adapt and test the proposed metaheuristics on other problems and challenges from the domain of WSNs as well. Moreover, we will also implement and improve other swarm approaches and adapt them for tackling the same an similar problems that should be addressed in WSNs implementation.

Since many challenges from the WSNs research area belong to the group of NP hard optimization, and due to the fact that the swarm intelligence metaheuristics proved to be robust methods for solving these kinds of problems, this area of research is prosperous and many opportunities for improvements exist. First, we will try to adapt the dynsTGA and the HEHO metaheuristics, which are proposed in this paper, for other WSNs problems, such as energy-based localization, deployment, coverage, and routing. Moreover, we also plan to perform experiments with different kinds of WSNs, like indoor, under water, under ground, and networks with mobile sensor nodes.

On the other hand, in the swarm intelligence research domain, there are also many opportunities for improvements. In general, swarm algorithms may be improved by applying minor and/or major changes/modifications. Minor improvements may be implemented by changing the value or behavior of control parameters, and/or changing some components of the exploitation or exploration search equations. Major improvements are in general performed by applying hybridization with other algorithms or metaheuristics.

In this context, we also plan to conduct research on existing swarm intelligence approaches improvements, and to adapt them to different kinds of WSNs challenges. When WSNs and swarm intelligence are combined, a huge research potential exists and this potential should be exploited and investigated.

## Figures and Tables

**Table 1:** Benchmark function details.

ID	Name of the Problem	Dim.	Type	Parameter Range	Optimum
F1	Ackley’s Problem (ACK)	10	Multimodal	(−30,30)	$f(x^{*}=0$) at $x^{*}=\left(0,\ldots ,0\right)$
F2	Aluffi–Pentini’s Problem (AP)	2	Multimodal	(−10,10)	$f(x^{*}\approx -0.3523$) at $x^{*}=\left(-1,\ldots ,0\right)$
F3	Becker and Lago Problem (BL)	2	Multimodal	(−10,10)	$f(x^{*}=0$) at $x^{*}=\left(-5,\ldots ,5\right)$
F4	Easom Problem (EP)	2	Unimodal	(−10,10)	$f(x^{*}=-1$) at $x^{*}=\left(\pi ,\pi \right)$
F5	Rastrigin Problem (RG)	10	Multimodal	(−5.12,5.12)	$f(x^{*}=0$) at $x^{*}=\left(0,\ldots ,0\right)$
F6	Rosenbrock Problem (RB)	10	Multimodal	(−30,30)	$f(x^{*}=0$) at $x^{*}=\left(1,\ldots ,1\right)$
F7	Goldstein and Price Problem (GP)	2	Multimodal	(−2,2)	$f(x^{*}=3$) at $x^{*}=\left(0,-1\right)$
F8	Gulf Research Problem (GRP)	2	Unimodal	(0,100)	$f(x^{*}=0$) at $x^{*}=\left(50,25,1.5\right)$

**Table 2:** Comparative analysis—tree growth algorithm (TGA) vs. dynamic search TGA (dynsTGA) (best results for each performance indicator are marked bold).

ID	Indicator	TGA	dynsTGA
	Best	0.00	0.00
F1	Mean	0.00	0.00
	StdDev	0.00	0.00
	Best	−0.35239	−0.35239
F2	Mean	−0.35239	−0.35239
	StdDev	6.09 × 10^−7^	**3.53 × 10^−9^**
	Best	1.07 × 10^−8^	**0.00**
F3	Mean	3.70 × 10^−7^	**2.04 × 10^−9^**
	StdDev	5.84 × 10^−7^	**5.11 × 10^−10^**
	Best	−0.99999	−0.99999
F4	Mean	−0.99999	−0.99999
	StdDev	1.59 × 10^−6^	**8.45 × 10^−7^**
	Best	0.00	0.00
F5	Mean	0.00	0.00
	StdDev	0.00	0.00
	Best	0.5653	**0.5361**
F6	Mean	**0.8231**	0.8502
	StdDev	0.3419	**0.3137**
	Best	3.00207	**3.00000**
F7	Mean	3.11253	**3.00068**
	StdDev	1.34 × 10^−1^	**7.305 × 10^−4^**
	Best	1.52 × 10^−5^	**8.45 × 10^−6^**
F8	Mean	7.05 × 10^−2^	**3.50 × 10^−2^**
	StdDev	2.60 × 10^−1^	**1.72 × 10^−1^**

**Table 3:** Comparative analysis—elephant herding optimization (EHO) vs. hybridized EHO (HEHO). (best results for each performance indicator are marked bold).

ID	Indicator	EHO	HEHO
	Best	1.3 × 10^−3^	**0.00**
F1	Mean	4.5 × 10^−2^	**0.00**
	StdDev	5.7 × 10^−5^	**0.00**
	Best	−0.35239	−0.35239
F2	Mean	−0.35239	−0.35239
	StdDev	5.13 × 10^−4^	**2.28 × 10^−8^**
	Best	5.05 × 10^−6^	**0.00**
F3	Mean	5.61 × 10^−3^	**5.44 × 10^−8^**
	StdDev	6.15 × 10^−5^	**6.01 × 10^−9^**
	Best	−0.99999	−0.99999
F4	Mean	−0.99999	−0.99999
	StdDev	5.56 × 10^−4^	**1.22 × 10^−5^**
	Best	0.00	0.00
F5	Mean	0.00	0.00
	StdDev	0.00	0.00
	Best	0.5792	**0.5395**
F6	Mean	0.8962	**0.8876**
	StdDev	0.3638	**0.3053**
	Best	3.00357	**3.00000**
F7	Mean	3.12002	**3.00083**
	StdDev	1.34 × 10^−1^	**6.211 × 10^−4^**
	Best	4.32 × 10^−5^	**2.05 × 10^−6^**
F8	Mean	**1.15 × 10^−2^**	4.64 × 10^−2^
	StdDev	**1.88 × 10^−1^**	1.92 × 10^−1^

**Table 4:** Comparative analysis—HEHO and dynsTGA vs. other metaheuristics for unconstrained benchmarks. (best results for each performance indicator are marked bold).

ID	Indicator	IBPA	LADA	TS	WSA	HEHO	dynsTGA
	Best	0.00815	0.00088	0.14185	0.888 × 10^−15^	0.00	0.00
F1	Mean	0.02260	0.00473	0.38528	0.888 × 10^−15^	0.00	0.00
	StdDev	0.01021	0.00157	0.07488	1.0029 × 10^−31^	0.00	0.00
	Best	−0.35238	−0.35238	−0.35238	−0.35238	−0.35239	−0.35239
F2	Mean	−0.35238	−0.35238	−0.35238	−0.35236	−0.35239	−0.35239
	StdDev	1.067 × 10^−6^	5.576 × 10^−7^	2.183 × 10^−5^	8.761 × 10^−6^	2.28 × 10^−8^	**3.53 × 10^−9^**
	Best	3.217 × 10^−9^	1.259 × 10^−9^	3.955 × 10^−7^	5.589 × 10^−8^	0.00	0.00
F3	Mean	2.826 × 10^−7^	2.486 × 10^−7^	7.637 × 10^−6^	1.267 × 10^−7^	5.44 × 10^−8^	**2.04 × 10^−9^**
	StdDev	2.838 × 10^−7^	2.704 × 10^−7^	6.302 × 10^−6^	3.877 × 10^−8^	6.01 × 10^−9^	**5.11 × 10^−10^**
	Best	−0.99999	−0.99999	−0.99999	−0.99999	−0.99999	−0.99999
F4	Mean	−0.83334	−0.99999	−0.46667	−0.99957	−0.99999	−0.99999
	StdDev	0.379010	**2.885 × 10^−6^**	0.507330	2.025 × 10^−4^	1.22 × 10^−5^	8.45 × 10^−5^
	Best	0.08790	0.00606	4.58753	0.00	0.00	0.00
F5	Mean	0.29275	0.01584	6.35541	0.00	0.00	0.00
	StdDev	0.12481	0.00554	0.89405	0.00	0.00	0.00
	Best	1.6578	13.1161	24.7395	8.9167	0.5395	**0.5361**
F6	Mean	12.1420	26.4740	66.1024	8.9449	0.8876	**0.8502**
	StdDev	14.9202	14.9521	19.1763	0.0160	**0.3053**	0.3137
	Best	3.00000	3.00000	3.00000	3.00000	3.00000	3.00000
F7	Mean	5.70001	10.00710	3.00053	**3.00032**	3.00083	3.00068
	StdDev	8.23847	16.46670	5.751 × 10^−4^	1.622 × 10^−4^	6.211 × 10^−4^	**7.305 × 10^−4^**
	Best	5.399 × 10^−6^	8.124 × 10^−5^	3.120 × 10^−5^	32.83	2.05 × 10^−6^	**8.45 × 10^−6^**
F8	Mean	**0.00157**	5.362 × 10^−4^	2.047 × 10^−4^	32.83	4.64 × 10^−2^	3.50 × 10^−2^
	StdDev	0.00162	3.456 × 10^−4^	1.382 × 10^−4^	**1.445 × 10^−15^**	1.92 × 10^−1^	1.72 × 10^−1^

**Table 5:** Simulation results for M=40, N=8 and the search domain area 100U×100U with different values for $P_{n}$ averaged in 30 runs—comparative analysis between EHO, HEHO, TGA, and dynsTGA (best results for each performance indicator are marked bold).

Algorithms	$P_{n}=5$			$P_{n}=2$		
	Mean $N_{\mathrm{NL}}$	Mean $E_{L}$	Computing Time (s)	Mean $N_{\mathrm{NL}}$	Mean $E_{L}$	Computing Time (s)
**EHO**	6.8	0.79	1.1	6.2	0.71	0.9
**HEHO**	5.3	0.45	1.2	5.1	0.37	1.0
**TGA**	5.5	0.42	**0.9**	5.0	0.36	**0.8**
**dynsTGA**	**4.5**	**0.19**	1.2	**4.3**	**0.16**	1.1

**Table 6:** Simulation results for M=40, N=8 and the search domain area 100U×100U with different values for $P_{n}$ averaged in 30 runs—comparative analysis between butterfly optimization algorithm (BOA), firefly algorithm (FA), particle swarm optimization (PSO), EHO, HEHO, TGA, and dynsTGA (results for BOA, FA, and PSO were taken from [15]) (best results for each performance indicator are marked bold).

Algorithms	$P_{n}=5$			$P_{n}=2$		
	Mean $N_{\mathrm{NL}}$	Mean $E_{L}$	Computing Time (s)	Mean $N_{\mathrm{NL}}$	Mean $E_{L}$	Computing Time (s)
**BOA**	4.7	0.28	0.65	4.5	0.21	0.53
**FA**	6.6	0.72	2.15	6.2	0.69	1.94
**PSO**	5.9	0.81	0.54	5.6	0.78	0.49
**EHO**	6.8	0.79	1.1	6.2	0.71	0.9
**HEHO**	5.3	0.45	1.2	5.1	0.37	1.0
**TGA**	5.5	0.42	0.9	5.0	0.36	0.8
**dynsTGA**	**4.5**	**0.19**	1.2	**4.3**	**0.16**	1.1

**Table 7:** Detailed results of EHO, HEHO, TGA, and dynsTGA proposed localization ($N_{L}$ = number of localized nodes, $E_{L}$ = localization error, $T_{E}$ = execution time in seconds).

Target Node	Anchor Node	Trial		EHO			HEHO			TGA			dynsTGA	
			$N_{L}$	$E_{L}$	$T_{E}$	$N_{L}$	$E_{L}$	$T_{E}$	$N_{L}$	$E_{L}$	$T_{E}$	$N_{L}$	$E_{L}$	$T_{E}$
25	10	1	17	0.654592	0.83	21	0.305502	1.15	20	0.273054	0.79	25	0.195331	1.36
		2	21	0.756329	1.05	22	0.278037	0.98	23	0.295530	0.65	23	0.168431	1.22
		3	18	0.776231	0.89	21	0.246762	1.03	22	0.227991	0.87	25	0.177892	1.39
		4	21	0.695520	1.11	20	0.239952	1.16	19	0.309982	0.92	24	0.209644	1.16
		5	18	0.685976	0.95	19	0.257405	0.99	20	0.251982	0.73	25	0.195799	1.41
50	15	1	47	0.473320	1.32	49	0.395029	1.73	50	0.314875	1.32	47	0.275583	1.96
		2	46	0.399921	1.53	50	0.365282	1.85	48	0.263050	1.17	46	0.219592	2.13
		3	50	0.635486	1.49	46	0.449252	1.59	46	0.430058	1.41	50	0.259765	1.86
		4	47	0.370542	1.55	49	0.269440	1.93	47	0.305406	1.49	47	0.230059	2.06
		5	49	0.556254	1.58	50	0.424532	1.66	48	0.253679	1.22	48	0.295904	2.16
75	20	1	74	0.639521	2.33	72	0.350875	2.20	74	0.263231	1.65	73	0.199861	2.62
		2	70	0.558261	1.95	74	0.280843	2.73	75	0.386699	2.07	75	0.180029	2.99
		3	73	0.725201	2.16	75	0.273232	2.16	74	0.235611	1.83	75	0.219865	2.70
		4	75	0.592035	2.43	71	0.405024	2.66	70	0.240959	1.66	72	0.299400	2.56
		5	72	0.773061	2.37	74	0.301557	2.27	73	0.334012	2.22	72	0.160293	2.51
100	25	1	99	0.538522	2.52	100	0.414563	3.03	100	0.322105	2.72	100	0.225200	3.61
		2	100	0.660631	3.07	99	0.295402	3.52	100	0.288644	2.69	100	0.260502	3.15
		3	97	0.502085	2.87	99	0.289436	3.89	100	0.437275	2.16	100	0.194039	4.11
		4	100	0.527651	2.90	99	0.500929	3.16	100	0.290011	2.92	100	0.246309	3.62
		5	96	0.685542	2.44	100	0.305521	3.44	100	0.400091	3.06	100	0.205582	4.01
125	30	1	124	0.832529	3.81	122	0.605331	5.05	125	0.529099	3.75	125	0.445044	4.82
		2	120	0.602132	4.22	125	0.633079	4.24	123	0.675249	3.26	125	0.575166	4.95
		3	121	0.919308	4.38	124	0.999975	5.13	125	0.561629	3.18	124	0.398802	5.75
		4	125	0.762035	3.67	123	0.675226	4.29	123	0.530555	3.66	125	0.609182	4.52
		5	121	0.613552	4.31	125	0.660022	4.95	124	0.607011	3.79	124	0.399805	5.55
150	35	1	149	0.899913	5.71	150	0.655520	6.85	149	0.870252	4.71	149	0.621203	7.43
		2	148	0.727201	4.69	150	0.893529	5.75	150	0.730318	4.33	150	0.825276	6.06
		3	150	0.708222	5.37	149	0.698736	6.71	150	0.690091	4.79	150	0.815900	6.23
		4	150	0.966152	5.03	150	0.851012	5.69	149	0.655213	4.28	149	0.599972	7.33
		5	148	0.698657	4.81	149	0.889902	6.66	149	0.820510	4.79	150	0.872217	6.77

**Table 8:** Detailed results of BOA, FA, PSO, HEHO, and dynsTGA proposed localization—results for BOA, FA, and PSO were taken from [15] ($N_{L}$ = number of localized nodes, $E_{L}$ = localization error, $T_{E}$ = execution time in seconds).

Target Node	Anchor Node	Trial		BOA			FA			PSO			HEHO			dynsTGA	
			$N_{L}$	$E_{L}$	$T_{E}$	$N_{L}$	$E_{L}$	$T_{E}$	$N_{L}$	$E_{L}$	$T_{E}$	$N_{L}$	$E_{L}$	$T_{E}$	$N_{L}$	$E_{L}$	$T_{E}$
25	10	1	23	0.207908	0.40	19	0.335551	1.44	22	0.807158	0.36	21	0.305502	1.15	25	0.195331	1.36
		2	24	0.188224	0.33	20	0.246423	1.44	17	0.728214	0.39	22	0.278037	0.98	23	0.168431	1.22
		3	25	0.224510	0.38	21	0.296398	1.70	18	0.797650	0.40	21	0.246762	1.03	25	0.177892	1.39
		4	25	0.19963	0.38	20	0.256168	1.65	17	0.739102	0.39	20	0.239952	1.16	24	0.209644	1.16
		5	24	0.212247	0.31	19	0.278459	1.57	19	0.799164	0.36	19	0.257405	0.99	25	0.195799	1.41
50	15	1	46	0.235326	0.77	50	0.505511	2.50	48	0.578797	0.74	49	0.395029	1.73	47	0.275583	1.96
		2	49	0.260490	0.81	49	0.326980	4.42	50	0.753254	0.85	50	0.365282	1.85	46	0.219592	2.13
		3	48	0.361080	0.92	49	0.254824	1.63	47	0.587004	0.75	46	0.449252	1.59	50	0.259765	1.86
		4	50	0.323910	0.86	48	0.227842	3.90	46	0.438748	0.76	49	0.269440	1.93	47	0.230059	2.06
		5	49	0.351415	0.91	49	0.2476413	4.19	47	0.486784	0.85	50	0.424532	1.66	48	0.295904	2.16
75	20	1	75	0.328310	1.68	74	0.703964	2.97	75	0.67414	1.31	72	0.350875	2.20	73	0.199861	2.62
		2	75	0.219680	1.52	75	0.291862	2.73	75	0.720123	1.35	74	0.280843	2.73	75	0.180029	2.99
		3	68	0.178960	1.52	72	0.279126	5.84	73	0.771325	1.30	75	0.273232	2.16	75	0.219865	2.70
		4	75	0.183942	1.43	71	0.284865	4.70	72	0.798457	1.32	71	0.405024	2.66	72	0.299400	2.56
		5	73	0.196781	1.69	73	0.2907846	3.97	73	0.697814	1.31	74	0.301557	2.27	72	0.160293	2.51
100	25	1	100	0.218838	2.29	100	0.779716	5.66	100	0.668227	2.49	100	0.414563	3.03	100	0.225200	3.61
		2	100	0.295008	2.27	100	0.299194	6.33	100	0.614843	2.10	99	0.295402	3.52	100	0.260502	3.15
		3	100	0.216414	2.32	100	0.385758	3.55	100	0.608155	2.20	99	0.289436	3.89	100	0.194039	4.11
		4	100	0.235804	2.37	100	0.589494	4.56	100	0.627197	2.35	99	0.500929	3.16	100	0.246309	3.62
		5	100	0.259312	2.45	100	0.513591	4.93	100	0.653258	2.16	100	0.305521	3.44	100	0.205582	4.01
125	30	1	124	0.615712	3.12	122	0.938894	2.707	119	0.600957	3.90	122	0.605331	5.05	125	0.445044	4.82
		2	123	0.437651	3.65	123	0.651459	5.995	123	0.662322	3.87	125	0.633079	4.24	125	0.575166	4.95
		3	125	0.568754	4.26	123	0.831683	2.709	125	0.593421	4.90	124	0.999975	5.13	124	0.398802	5.75
		4	124	0.657499	3.76	125	0.950842	3.11	125	0.608412	3.98	123	0.675226	4.29	125	0.609182	4.52
		5	125	0.545789	3.87	125	0.912666	5.894	125	0.744193	4.90	125	0.660022	4.95	124	0.399805	5.55
150	35	1	150	0.743780	5.67	149	0.957818	3.386	149	0.657679	5.03	150	0.655520	6.85	149	0.621203	7.43
		2	150	0.887561	4.87	150	0.973891	3.459	149	0.773764	5.16	150	0.893529	5.75	150	0.825276	6.06
		3	150	0.765347	5.65	150	0.854096	5.894	150	0.620403	4.22	149	0.698736	6.71	150	0.815900	6.23
		4	149	0.665348	4.12	150	0.672451	4.87	150	0.766621	5.21	150	0.851012	5.69	149	0.599972	7.33
		5	150	0.787689	4.76	120	0.632727	3.356	150	0.625278	4.43	149	0.889902	6.66	150	0.872217	6.77
